# Does brand community participation lead to early new product adoption? The roles of networking behavior and prior purchase experience

**DOI:** 10.3389/fpsyg.2023.1014825

**Published:** 2023-03-08

**Authors:** Ying Jiang, Junyun Liao, Jiecong Pang, Hsin-Li Hu

**Affiliations:** ^1^Economics and Management School, Wuhan University, Wuhan, China; ^2^Research Institute on Brand Innovation and Development of Guangzhou, School of Management, Jinan University, Guangzhou, China; ^3^School of Communication, The Hang Seng University of Hong Kong, Hong Kong, Hong Kong SAR, China

**Keywords:** brand community, social networks, in-degree centrality, out-degree centrality, new product adoption brand community, new product adoption

## Abstract

**Introduction:**

Consumers’ adoption behavior is critical to the success of new products, but the effects of brand communities on new product adoption have rarely been investigated. In this study, we draw on network theory to examine how consumer participation in brand communities (in terms of participation intensity and social networking behaviors) affects the adoption of new products.

**Methods:**

We collected longitudinal data from 8,296 members of an online community of a well-known smartphone brand to assess the factors influencing new product adoption.

**Results:**

The results from applying a hazard model indicated that brand community participation increases the speed of adoption of new products. The positive effect of members’ out-degree centrality on new product adoption was found to be significant, but in-degree centrality only had an effect when users had previous purchasing experience.

**Discussion:**

These findings extend the literature by revealing how new products are disseminated across brand communities. The study also makes theoretical and practical contributions to the literature on brand community management and product marketing.

## Introduction

In response to constant advances in technology and intense competition, firms must quickly develop and launch new products to ensure that the new demands of consumers are met (Cooper, 2019; Liao et al., 2023; Veloutsou and Liao, 2023; Zheng et al., 2023). The launch of a new product strongly depends on its rapid adoption by consumers after its launch (Nguyen and Chaudhuri, 2019). Choosing new and unfamiliar products can involve a high level of uncertainty. To alleviate such feelings, consumers will search for information such as word-of-mouth recommendations from friends (Peres et al., 2010; Rogers, 2010), particularly for technological products (e.g., smartphones) that have short lifetimes and lose value quickly (Liao et al., 2021a,b,c,d). In the traditional media context, consumers are relatively isolated, as peer-to-peer interactions are limited (Kim and Chandler, 2018), but through social media, they can easily interact and communicate with each other *via* brand communities (Rapp et al., 2013; Lamberton and Stephen, 2016; Wang et al., 2021). Consumers’ identities then become associated with their community membership (Thompson and Sinha, 2008; Liao et al., 2020a), and their social networks and past purchasing experiences jointly influence their adoption of new products.

Research has suggested that a brand community can effectively facilitate new product adoption. First, communities can enhance consumers’ brand loyalty (Zheng et al., 2015; Jibril et al., 2019; Liao et al., 2020b), which can encourage them to purchase a product (Chi, 2018). Second, brand communities can serve as information disseminators when new products are being developed (Gruner et al., 2014), which can effectively encourage their adoption (Rogers, 2010). Third, the social influences that brand communities have on their members can vary (Algesheimer et al., 2005), and a positive long-term peer effect can influence their attitudes toward new products (Bailey et al., 2022).

Studies have suggested that participation in a brand community increases the likelihood that consumers will buy new products from the brand rather than from its competitors (Thompson et al., 2018). Most studies have focused on the relationships between community participation and variables such as community identification and brand loyalty (Pai and Tsai, 2011; Hook et al., 2018; Thompson et al., 2019). However, community network structures and the social relationships between members can also affect consumers’ adoption of new products (Yan et al., 2014). Studies have also shown that past purchasing experiences influence consumers’ adoption behavior (Rahman and Mannan, 2018; Jia et al., 2021), but not in the context of brand communities. To address this research gap, we collected data from the online community of a global smartphone brand (Samsung) to examine how brand community participation, social relations (i.e., in-degree and out-degree centrality), and purchase experiences influence consumers’ adoption of new products. Following previous studies, we first defined in-degree centrality and out-degree centrality in the context of brand communities (Jarvinen and Nicholls, 1996; van den Bulte et al., 2007; Hu et al., 2015) and then examined the relationships between all of the constructs. In-degree centrality is defined as the number of community members who follow a focal member in a brand community, and out-degree centrality is the number of members the focal member is following. The results of a hazard model indicated that brand community participation had a positive and significant effect on new product adoption, which was positively moderated by purchasing experience. We found that out-degree centrality had a positive effect on new product adoption, while the effect of in-degree centrality was only apparent when consumers had extensive purchasing experience.

This article extends the literature by considering social relations and the moderating role of purchase experience in terms of new product adoption. Network theory has long been applied in the marketing literature (Akar and Dalgic, 2018; Ebrahimi et al., 2022), but the network structures of brand communities have rarely been studied (Lee et al., 2011). Our findings offer a new perspective on the effect of brand communities and provide practical suggestions for how managers can improve their marketing strategies for new products. In this study, we first briefly review the conceptual background of the brand community literature and network theory and then propose our hypotheses and introduce the research methodology and findings. Finally, we present the theoretical contributions and managerial implications of this study, along with its limitations.

## Conceptual background

### Brand community and new product adoption

The development of the Internet and the proliferation of mobile devices enables consumers to gather in virtual communities and interact with each other based on their shared love of a brand, and subsequently to form a structured set of social relationships. These specialized, non-geographically based brand communities (Muniz and O’Guinn, 2001; Thompson et al., 2019) can reduce consumer uncertainty about purchasing decisions and facilitate the success of new products (Gruner et al., 2014; Liao et al., 2021a,b,c,d, 2022a,b) even if they underperform relative to competitors’ offerings (Thompson et al., 2018). Communities activate brand loyalty and create a sense of oppositional loyalty (Zhang et al., 2016; Coelho et al., 2018; Sohail et al., 2020; Liao et al., 2021a,b,c,d), serve as channels for disseminating and sharing information (Kim et al., 2008; Wang et al., 2019), and create a peer social influence effect among members (Algesheimer et al., 2005; Hook et al., 2018).

First, various studies have indicated that brand communities play an important role in enhancing brand loyalty (Zhang et al., 2016; Coelho et al., 2018; Sohail et al., 2020; Fathy et al., 2022; Samarah et al., 2022). Interaction and participation in these communities can help establish brand loyalty (Casaló et al., 2010; Liao et al., 2017), as can identification with a community (Kaur et al., 2020; Dessart and Veloutsou, 2021; Deng et al., 2023) and commitment to it (Hur et al., 2011; Bao and Wang, 2021). Loyalty positively affects consumers’ purchasing behavior in terms of new products (Chi, 2018). Community membership leads to a sense of oppositional loyalty (Kuo and Feng, 2013; Liao et al., 2021a,b,c,d) by encouraging members to avoid using products from rival brands (Thompson and Sinha, 2008).

Second, brand communities can serve as communication channels through which information about new products can be transmitted (Kim et al., 2008; Wang et al., 2019). Community managers provide information about new products offered by the brand (Iyengar et al., 2011) and selectively expose community members to this information (Thompson and Sinha, 2008). The members also disseminate and share product information across the membership and with the public (Gruner et al., 2014). This helping behavior allows members to learn about each other’s purchasing experiences and share product knowledge (Liao et al., 2022a,b). As Rogers (2010) noted, the dissemination of new product information facilitates consumers’ purchasing intentions.

Third, various social factors can influence brand community members (Algesheimer et al., 2005; Hook et al., 2018), including the influence of peers on adoption behavior. If an individual’s friends purchase a product, the likelihood that the individual will buy the product increases (Bhatt et al., 2010; Eggers et al., 2022). This positive influence has been found to be sustained over the long term (Bailey et al., 2022). Also, the interest in a new product of an individual’s friends and product-related information they shared can enhance the individual’s purchase intention (Chang and Cheng, 2016).

Thus, studies have suggested that a brand community can exert a positive influence on new product adoption by considering the joint impact of brand loyalty, information dissemination, and peer effect. Although the causal relationships between brand community participation and new product adoption have been established (Thompson and Sinha, 2008), few studies have examined the drivers of new product adoption behavior and the moderating effects of consumer characteristics, such as their networks and purchasing experiences. Therefore, further empirical investigation is needed in the brand community context (Cheng and Shiu, 2020).

### Network theory

Network theory has been applied to various marketing research areas, such as word-of-mouth behavior (Brown et al., 2007; Kozinets et al., 2010; Donthu et al., 2021), product adoption (Katona et al., 2011; Hinz et al., 2014), brand preferences (Ward and Reingen, 1990), information acquisition (Granovetter, 1973), and innovation performance (Carnabuci and Diószegi, 2015; Karamanos, 2016). Network theory suggests that individuals are embedded within their social relationships (Borgatti et al., 2009). These relationships generate tangible and intangible benefits and valuable resources for the focal actor (i.e., the ego) and constrain individual behavior within the roles defined by these relationships (Krackhardt, 1999; Gargiulo and Benassi, 2000). Network theory has been applied to assess the relationships between brand community members (Katz et al., 2018) and to better explain their consumption behavior (Katz et al., 2020). For instance, Lee et al. (2011) has applied network theory to analyze the operations of brand communities and examine the influences a network’s structural characteristics have on members’ emotional attachment toward the community.

The structure of a network consists of nodes and links, where each node denotes a member within the network and each link a relationship between the adjacent nodes (Lee et al., 2011). The relationships between community members can thus be described through this type of structure, so network theory is appropriate for analyzing brand communities. Consistent with the traditional view of social networks (Nahapiet and Ghoshal, 1998; Park and Cho, 2012; Kumar and Zaheer, 2019), we use ego network characteristics as a proxy for measuring member-to-member relationships in a brand community. These constitute the horizontal relationships of members. Ego networks include the characteristics of in-degree and out-degree centrality, in which in-degree centrality is defined as the number of links pointing inward toward a node and out-degree centrality as the number of links pointing outward to other nodes (Hansen et al., 2011). We follow Hu et al. (2015) and argue that we can use in-degree centrality to assess the level of acceptance or popularity of a brand community member and out-degree centrality to identify a member’s information sources. In-degree centrality thus measures the number of community members who follow a focal member in a brand community. This illustrates the focal member’s popularity and can be understood as a sociometric reflection of an individual’s attractiveness, which can fulfill their need for relatedness (Jarvinen and Nicholls, 1996). High in-degree centrality can lead to group receptivity, elevated status, popularity, and prominence for the member and enhance their self-esteem (Bonacich, 1987; Kwon and Ha, 2023). Any information generated by the member can also be received by more community members (Brown and Reingen, 1987; Gibbons and Olk, 2003; Yang et al., 2018). Out-degree centrality measures the number of members a focal member is following in a brand community (van den Bulte et al., 2007). High out-degree centrality indicates that the focal member receives information from many sources and reflects the level of trust the focal member has in other members. As Longobardi et al. (2020) established, these two variables are independent.

## Research model and hypotheses

### Community participation and new product adoption over time

Brand community participation refers to members’ interactions within such a community (Tsai et al., 2012). Studies have indicated that brand community participation directly stimulates members’ purchasing intentions (Cheung et al., 2015; Ho, 2015; Kumar and Nayak, 2019) and facilitates their brand loyalty (Madupu and Cooley, 2010; Lin et al., 2011; Liao et al., 2021a,b,c,d), which increases their intentions to adopt new products (Chi, 2018). Participation in brand communities can overcome the switching costs by fostering members’ attachment to the brand’s products, which arises from the product compatibility problems and significantly reduce the likelihood that consumers will adopt new products (Thompson et al., 2019). By providing information about new products, these communities can reduce any uncertainties consumers may have (Adjei et al., 2010; Stock et al., 2021), thus encouraging them to adopt new products. Thus, consumer participation in brand community activities has been found to significantly enhance their willingness to buy new products. We therefore propose the following:

*H1*: Brand community members with a higher level of community participation are more likely to adopt new products earlier.

### Community members’ ego networks and new product adoption

Individuals’ levels of in-degree centrality, that is, the number of incoming links they have in their social network, can play a role in satisfying their need for social connectedness with other people (Valente et al., 2008; Musiał et al., 2009; Lin, 2016). This reflects their popularity as perceived by other members and can enhance their self-esteem by providing recognition and status (van den Bulte et al., 2007; Lee et al., 2010; Fernández-Zabala et al., 2020). In-degree centrality can therefore have various effects on new product adoption. First, as De Bruyn and Van Den Boom (2005) and Lee et al. (2010) have noted, a member’s popularity is positively related to their self-esteem, which is in turn positively related to their intention to purchase (Sierra et al., 2016). Thus, community members with a high level of in-degree centrality may adopt a new product earlier than other members. Second, members’ popularity provides them with social support (Hashim and Tan, 2015; Tajvidi et al., 2021), reducing concerns that arise about purchasing a new product and enhancing the anticipatory pleasure derived from using it (Thompson et al., 2019). Third, high in-degree centrality suggests that a member is trusted by others and quite influential in the community (Cross and Cummings, 2004; Lee et al., 2011). Such opinion leaders can thus accelerate the adoption of a product by other members of the social network (Lin et al., 2018; Zhang and Gong, 2021). A member with influence in a community network will also be more attached to the focal brand (Lee et al., 2011), and brand attachment has been found to be positively related to consumers’ purchase intentions (Gilal et al., 2021; Petravičiūtė et al., 2021). Thus, consumers with high in-degree centrality will be more likely than those with low in-degree centrality to adopt a new product soon after it is launched. We therefore propose the following:

*H2*: The higher the in-degree centrality of a focal brand community member, the more likely the member will be to adopt the new product earlier than members with lower out-degree centrality.

We measure out-degree centrality by the number of other members a focal member is following in the community (i.e., their outgoing links). Out-degree centrality can affect when new products are adopted. First, a high level of out-degree centrality indicates that the focal member receives extensive information from many sources (Musiał et al., 2009; Lee et al., 2010). Information about the attributes of a new product increases its perceived value and thus the intention to purchase (Chang and Wildt, 1994). Information can also reduce uncertainty and the perceived risk perception of adopting the product (Chen et al., 2022). Second, network theory suggests that attitudes are not innate or developed in isolation (Erickson, 1988). Individual attitudes are mainly formed and changed through social interaction, so attitude similarity can arise through regular social interactions (Wan et al., 2017). Once a new product is launched, common attitudes about it emerge through social interactions between brand enthusiasts because of their tendency to quickly form similar positive attitudes toward a product. Thus, they are likely to adopt it earlier than others (Thompson and Sinha, 2008). This attitude similarity means that the information that members share is likely to originate from a similar source, which makes it more helpful and thus increases their purchasing intentions toward a new product (Filieri et al., 2018). Thus, we propose the following:

*H3*: The higher the out-degree centrality of a community member, the more likely the member will be to adopt the new product earlier than members with lower out-degree centrality.

### The moderating effect of purchasing experience

Purchasing experience refers to the previous purchasing of a brand, and has been found to significantly affect consumers’ future shopping behavior (Shim et al., 2001). Consumers form attitudes toward a new product based on their experience (Jacoby and Kyner, 1973; Ling et al., 2010). Research has found that purchasing experience enables consumers to search for product information more easily and weakens the effect of perceived risk (Li and Yuan, 2018). Uncertainty about new products is reduced, thus strengthening the positive influence of brand community participation on new product adoption. Studies have also indicated that purchasing experience can enhance consumers’ expertise in product knowledge (Rodgers et al., 2005), further enabling them to successfully search for and process information about new products (Hernández et al., 2010; Yoon, 2010). Thus, brand community participation can enhance new product adoption through the provision of information, and purchasing experience can increase this effect. We therefore propose the following:

*H4*: Purchasing experience positively moderates the relationship between brand community participation and new product adoption.

High in-degree centrality indicates an individual’s importance in a social network and denotes the position of opinion leader in a brand community (Eck et al., 2011; Cho et al., 2012). A previous study indicated that opinion leaders possess extensive knowledge about products and the market (Katz and Lazarsfeld, 2017), which they obtain *via* their purchasing experience (Rodgers et al., 2005). Therefore, purchasing experience can strengthen the positive effect of in-degree centrality on new product adoption by increasing the opinion leader’s professional knowledge of product. In addition, if consumers regularly have satisfactory experiences when purchasing from a particular brand, they will be optimistic about the brand and maintain their expectation of the brand’s high quality products (Mikulincer and Shaver, 2005), and will further develop brand attachment (Kang et al., 2017). A high level of in-degree centrality can therefore encourage the adoption of new products through facilitating brand attachment (Gilal et al., 2021; Petravičiūtė et al., 2021), and so purchasing experience can positively moderate this effect. We therefore propose the following:

*H5*: Purchasing experience positively moderates the relationship between in-degree centrality and new product adoption.

## Data and methodology

### Data

We obtained our data from the Galaxy Community, which was established by Samsung in March 2015. This brand community, in which users can communicate about Samsung’s mobile phones, attracted more than 5 million users in its first year. Registered users can generate content, browse posts, and comment on other users’ posts. Each user has a personal profile page that provides information on their community participation (including their posts, comments, followers, and who they follow) and general personal information including username, user ID, address and hobbies, gender, and product badges. Only usernames and user IDs are required, so only a few members choose to provide additional personal information. The community has a unique product badge system, in which purchased items are displayed on users’ profile pages along with the purchase date. Users must purchase Samsung phones and register their International Mobile Equipment Identity (IMEI) codes on the community website to obtain the corresponding badges. This product badge system enabled us to observe users’ product purchasing behavior.

Our focus was on the diffusion of a mobile phone product, the Samsung Galaxy Note 9, across the brand community. This phone was launched on August 15, 2018, but Samsung first offered a community sub-forum devoted to it in June 2018, probably to raise users’ interest in the product. Discussions about the product in the sub-forum were in the form of posts and comments. Thus, we extracted all user data from this sub-forum between June 2018 and April 2019, including users’ community participation, profile information, and product badges. Our sample included 8,296 users, of whom 1,848 had bought a Galaxy Note 9 by the end of the observation period.

#### Measures

##### Product adoption

As mentioned above, we determined whether a user bought the product by establishing whether they had a badge referring to it and the corresponding date of obtaining the badge. In-depth interviews with more than 50 users from the community revealed that almost all of them regarded it as an honor to obtain product badges, as these demonstrated their loyalty to Samsung. Virtual gifts, coupons, and service priority, which are provided to encourage them to purchase products, are very attractive to brand community members. Thus, the product badges reasonably reflect users’ actual adoption behavior. We therefore used the variable *Adoption* to indicate whether a user adopted the product by the end of the observation period. *Adoption_time* was measured as the number of days from the product release date to the adoption date or until the end date of the observation period if no purchase was made.

##### Participation

Participation was measured by the total number of posts and comments generated by a user until they bought the item or before the end of the observation period if they did not. This measure is widely used in the literature (Thompson and Sinha, 2008).

##### In-degree centrality

This was measured by a user’s number of followers. A high number indicated that a user was more popular and had a high level of in-degree centrality.

##### Out-degree centrality

The social ties between two users in a community are not necessarily bidirectional, as a user can follow others without their reciprocity or approval. Thus, we measured this variable by the number of other members a member followed during the observation period.

##### Purchase experience

We measured purchase experience by the number of other Samsung products a member purchased before the release of the Samsung Galaxy Note 9. Several control variables were also included in the model estimation.

##### Tribes

The Samsung community has many sub-forums, and users can participate in them simultaneously. Thus, we included how many sub-forums (i.e., tribes) a user participated in during the observation period.

The three variables of *participation*, *in-degree*, and *out-degree* demonstrated significant non-normality. To avoid a high level of skewness, we conducted a natural log transformation. Because of zero values in the data set, we also added a small positive number (0.1) to the measures before the log transformations (Butler and Wang, 2012). The summary statistics and correlations of the variables are provided in Tables 1, 2.

**Table 1:** Summary statistics.

Statistic	*N*	Mean	St. Dev.	Min	Max
Participation	8,296	−1.130	3.463	−4.605	8.320
In_degree	8,296	−3.089	2.728	−4.605	9.809
Purchase experience	8,296	1.472	1.773	0	42
Tribes	8,296	1.315	5.532	0	65
Out_degree	8,296	−2.949	2.650	−4.605	6.907
Adoption	8,296	0.264	0.441	0	1

**Table 2:** Correlation matrix.

	1	2	3	4	5	6
Adoption						
Adoption_Time	−0.879^***^					
Participation	0.032^**^	−0.026^*^				
In_degree	−0.002	−0.004	0.124^***^			
Purchase experience	−0.002	−0.077^***^	0.157^***^	0.106^***^		
Tribes	−0.069^***^	0.046^***^	0.236^***^	0.057^***^	0.149^***^	
Out_degree	−0.013	−0.005	0.344^***^	0.103^***^	0.077^***^	0.214^***^

^*^p < 0.1,^**^p < 0.05, ^***^p < 0.01.

### Model and estimation

**Figure 1:**
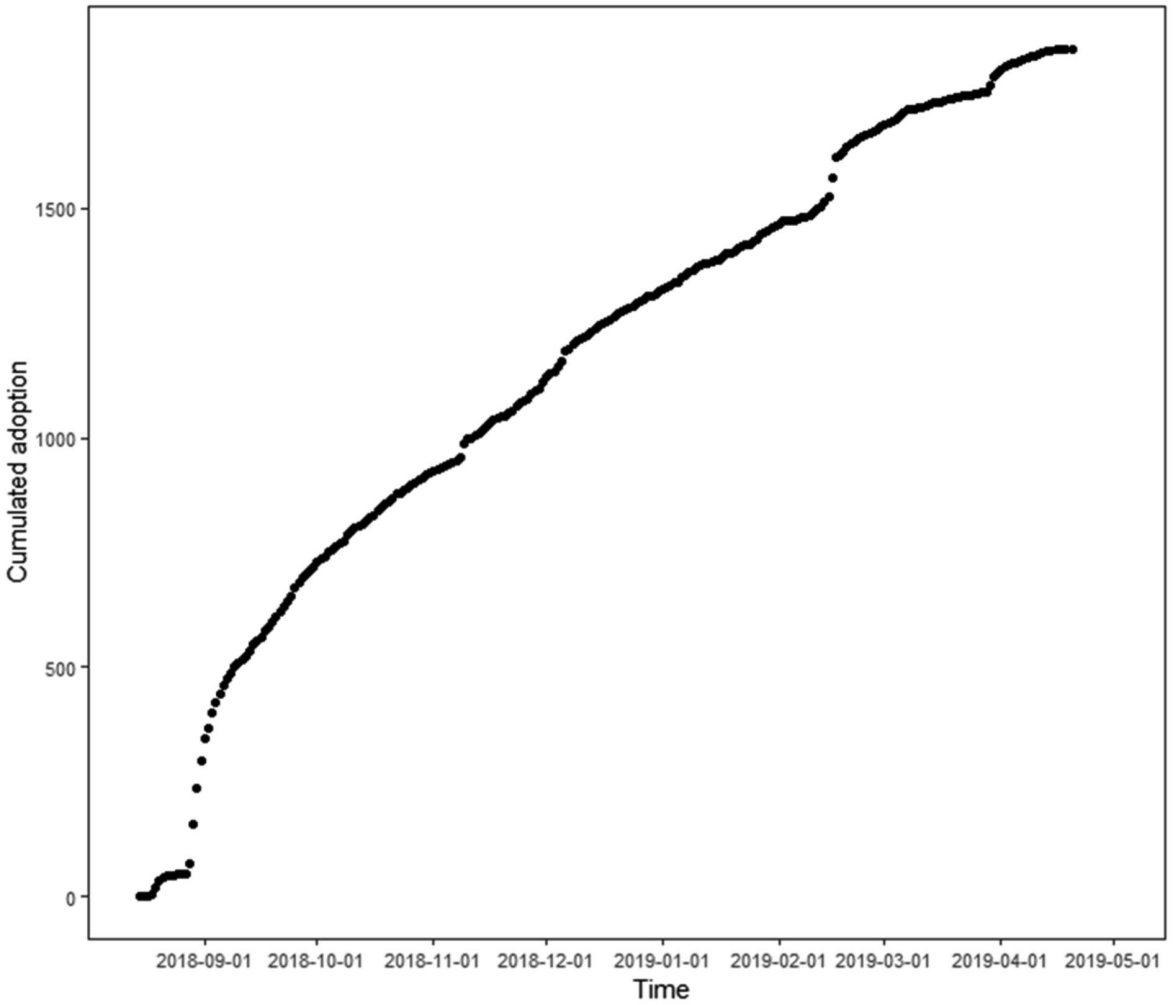
Product adoption over time.

Figure 1 visually presents the pattern of uptake of the Galaxy Note 9, indicating the accumulated level of adoption. The level initially increased rapidly and then slowed down. The shape of this pattern is significantly different from that of typical new product uptake, which is usually characterized by a slow and gradual increase. Consumers who participate in the brand community will typically have a strong preference for the brand, and those who directly discuss new products generally indicate that they are interested in them (Table 3).

**Table 3:** Hazard model.

	*Dependent variable:* Adoption_time	
	(1)	(2)
Participation	0.162^***^	0.108^***^
	(0.008)	(0.012)
In_degree	0.090	0.090
	(0.115)	(0.103)
Loyalty	0.039^**^	0.266^***^
	(0.016)	(0.036)
Tribes	−0.055^***^	−0.066^***^
	(0.010)	(0.011)
Out_degree	0.032^***^	0.029^***^
	(0.010)	(0.010)
Participation: Purchase experience		0.059^***^
		(0.009)
In_degree: Purchase experience		0.007^**^
		(0.003)
Observations	8,296	8,296
*R* ^2^	0.049	0.061
Log Likelihood	−19,024.050	−18,971.800
Wald Test	401.750^***^ (df = 5)	506.220^***^ (df = 7)
LR test	419.752^***^ (df = 5)	524.239^***^ (df = 7)
Score (log rank) test	422.591^***^ (df = 5)	515.492^***^ (df = 7)

^*^p < 0.1, ^**^p < 0.05, ^***^p < 0.01. Standard errors in parentheses.

Hazard modeling, a statistical technique for determining the probability that an individual will experience a specific event, was applied in this study to examine the relationship between brand community participation and the rate of adoption of new products. This approach enabled us to analyze the effects of various factors on product lifetime by using the rate of product adoption and the time of adoption as a factor variable. The duration can be considered as a random variable of the probability density f(t) and the cumulative distribution function F(t). The user’s adoption behavior is given a value of 1 if they purchase the product or 0 if they did not within the data collection period. We use h(t) to indicate the likelihood that a user will buy the product at time t. We assume that the basic rate for the risk that the user will not buy the product at time t is h_0_(t); therefore,


$$h\left(t\right)=h_{0}\left(t\right)\exp\left(\beta_{i}x_{i}\right)+\varepsilon_{i}$$

## Results

### Participation

The results indicated that participation was significantly correlated with new product adoption (*β* = 0.162^***^, *p* < 0.01). We found that community members with high levels of participation had a greater tendency than others to purchase new products, which helps create value for users and the company.

### In-degree

In-degree centrality was not found to be significant in new product adoption (*β* = 0.090, *p* > 0.1).

### Out-degree

This variable was found to significantly affect the purchasing of new products (*β* = 0.032^***^, *p* < 0.01), probably because of consumers’ enthusiasm for community participation.

### Participation*purchase experience

We found that the interaction between brand community participation and previous loyalty positively affected new product adoption (*β* = 0.059^***^, *p* < 0.01). Thus, our H4 was supported. This finding suggests that consumers are more likely to purchase a new product if they have a history of purchasing products from the specific brand and if they participate enthusiastically in the community.

### In-degree*purchase experience

The results indicated that the interaction between in-degree centrality and previous loyalty positively affected new product adoption (*β* = 0.007^**^, *p* < 0.05). Thus, our H5 was supported. This suggests that although in-degree centrality had no significant direct effect on product purchasing, it may motivate consumers to purchase if they have a history of purchasing products from the brand.

### Purchase experience

Purchase experience was significantly positively correlated with the adoption rate for new products (*β* = 0.039^**^, *p* < 0.05). Consumers’ purchasing experiences partly reflect their loyalty to the brand, and our results show that if users have previously bought other products from this brand, they are more likely to buy new products than users who have not.

### Tribes

The number of users was found to have little effect on the adoption of new products (*β* = −0.055^***^, *p* < 0.01). The numbers of tribes reflects user participation in the brand community in addition to their interests.

## Discussion and implications

### Theoretical contribution

Our research makes several contributions to the literature on brand communities. First, we offer a new perspective on how brand communities can influence consumers’ behavior regarding new products by examining the characteristics of the social networks within these communities. The impact of network centrality in brand communities has been examined (Yan et al., 2014; Katz et al., 2020), but research has mainly focused on the influence of network centrality on the relationships between consumers and brands such as in terms of consumer engagement (Sanders et al., 2019) and psychological ownership (Kuchmaner et al., 2019). Our study thus extends the literature by investigating the effect of network centrality on consumer behavior regarding new products. We did not find that in-degree centrality directly influenced new product adoption, and thus our original prediction that opinion leaders will adopt new products earlier than others was not supported (Iyengar et al., 2011). However, in-degree centrality had a positive effect if a user had previously purchased a product from the brand, implying that only members with sufficient purchasing experience can become true opinion leaders (Lyons and Henderson, 2005; Lin et al., 2018; Tobon and García-Madariaga, 2021) and will purchase new products earlier than others (Iyengar et al., 2011). However, out-degree centrality was found to have a positive effect on new product adoption, suggesting that members are more likely to adopt new products if they follow many other members. This result supports research suggesting that consumers with more social ties are more susceptible to social influence than those with fewer social ties (Centola, 2010; Harrigan et al., 2012).

Second, our study extends the literature on new product adoption by revealing how information on new products is disseminated through virtual brand communities, rather than through traditional physical marketing processes. Research has indicated that social media is critical to the success of new products (Wu et al., 2019), but few studies have examined the value of brand communities in terms of new product adoption (Thompson and Sinha, 2008). The marketing of new products is expected to be faster through a brand community, as members will by definition have a stronger relationship with the brand than non-members and will thus be more interested in it and its products (Algesheimer et al., 2005; Tsai and Bagozzi, 2014; Yuan et al., 2020). We confirm this assumption by identifying the positive effect of community participation on new product adoption. Our results also suggest that the number of connections that people have in a network and their characteristics will affect the speed of new product diffusion in the context of brand communities (Peres et al., 2010; Lee et al., 2011).

Third, this study reveals that purchasing experience influences new product adoption, which is a novel finding (Li and Yuan, 2018). We found that such experience strengthens the influence of brand community participation and in-degree centrality on new product adoption. The findings also increase our general understanding of in-degree centrality. Research has suggested that opinion leaders with high in-degree centrality tend to be early adopters of new products (Iyengar et al., 2011). Our results suggest that this may only be the case when they possess sufficient purchasing experience, as we only found a positive effect of in-degree centrality on new product adoption when the user had extensively purchased in the past.

### Managerial implications

Our findings offer several managerial implications about the marketing of new products. We found that the degree of brand community participation not only was positively correlated with the adoption of new products but also that it speeds up the adoption rate. Thus, when launching new products, marketing managers should encourage consumers to participate in the brand’s community. This can also reduce the likelihood that consumers purchase the products of rival brands, which helps the firm remain competitive (Thompson and Sinha, 2008). Interactions in brand communities can effectively reduce consumer uncertainty about new products (Adjei et al., 2010; Stock et al., 2021), so consumers should be encouraged to participate in such communities when a new product is released.

Predicting consumer behavior is notoriously difficult (Li and Zheng, 2020; Liao et al., 2022a,b). Identifying consumers who are more likely to purchase new products is therefore important, and large online brand communities such as the Galaxy Community are thus particularly useful. We found that consumers with purchasing experience were more likely to buy new Samsung products than those without purchasing experience. Those with previous purchasing experience are thus generally most likely to purchase a new product soon after its launch and should therefore be the focus of marketing activities. In addition, our finding that community members with high out-degree centrality are more likely to buy new products earlier can help brand community managers identify target consumers when trialing new products (Thompson and Sinha, 2008; Samuel et al., 2018; Zhang et al., 2021).

## Limitations and future research

Our study has some limitations that can be addressed in future research. First, although we revealed the positive effect of brand community participation on the adoption rate of new products, other factors may have effects. For example, consumer-consumer interaction and consumer-brand interaction may have a impact on consumers’ new adopotion (Wang, 2021; Samarah et al., 2022). Other factors such as brand-hosted offline activities and consumer innovativeness could aslo be examined in future studies (Seyed Esfahani and Reynolds, 2021; Jiang et al., 2022). Variables such as the frequency of interactions between managers and users in the brand community may also affect adoption rates and should therefore be explored. Second, we only examined the impact of degree centrality on new product adoption; other characteristics of brand community social networks such as closeness centrality and degree centralization (Lee et al., 2011; Golbeck, 2015) may also have effects. Thus, further exploring the characteristics of social networks in brand communities will be of benefit.

## Data availability statement

The raw data supporting the conclusions of this article will be made available by the authors, without undue reservation.

## Author contributions

YJ collected data, performed analysis, proposed the framework and wrote an early draft. H-LH, JL, and JP participated in writing and reviewing and editing the manuscript. H-LH and JL provided funding support. All authors contributed to the article and approved the submitted version.

## Funding

This project is supported by National Natural Science Foundation of China (NSFC) (72272061 and 71802097); The Ministry of Education of Humanities and Social Science project (22YJC630077); Philosophy and Social Sciences Planning Program of Guangzhou (2021GZYB05 and 2022JDGJ06); Research Institute on Brand Innovation and Development of Guangzhou (2021CS05); Jinan University Management School Funding Program (GY21013); and Institute for Enterprise Development, Jinan University, Guangdong Province (2021MYZD04 and 2020CP03).

## Conflict of interest

The authors declare that the research was conducted in the absence of any commercial or financial relationships that could be construed as a potential conflict of interest.

## Publisher’s note

All claims expressed in this article are solely those of the authors and do not necessarily represent those of their affiliated organizations, or those of the publisher, the editors and the reviewers. Any product that may be evaluated in this article, or claim that may be made by its manufacturer, is not guaranteed or endorsed by the publisher.
